# Management of mixed cryoglobulinemia with rituximab: evidence and consensus-based recommendations from the Italian Study Group of Cryoglobulinemia (GISC)

**DOI:** 10.1007/s10067-022-06391-w

**Published:** 2022-09-28

**Authors:** Luca Quartuccio, Alessandra Bortoluzzi, Carlo Alberto Scirè, Antonio Marangoni, Giulia Del Frate, Elena Treppo, Laura Castelnovo, Francesco Saccardo, Roberta Zani, Marco Candela, Paolo Fraticelli, Cesare Mazzaro, Piero Renoldi, Patrizia Scaini, Davide Antonio Filippini, Marcella Visentini, Salvatore Scarpato, Dilia Giuggioli, Maria Teresa Mascia, Marco Sebastiani, Anna Linda Zignego, Gianfranco Lauletta, Massimo Fiorilli, Milvia Casato, Clodoveo Ferri, Maurizio Pietrogrande, Pietro Enrico Pioltelli, Salvatore De Vita, Giuseppe Monti, Massimo Galli

**Affiliations:** 1 Unit of Rheumatology, Department of Medicine (DAME), University of Udine, ASUFC, Udine, Italy; 2 Section of Rheumatology, Department of Medical Sciences, University of Ferrara and Azienda Ospedaliera, Universitaria Di Ferrara, Cona, FE Italy; 3 Epidemiology Unit, Italian Society for Rheumatology (SIR), Milan, Italy; 4 Department of Internal Medicine, Hospital of Legnano, Legnano, Italy; 5 Medicina Interna, Ospedale Di Saronno, AO Busto Arsizio, Italy; 6 UO Nefrologia, Spedali Civili, Brescia, Italy; 7 ASUR Marche ZT6, Fabriano, Italy; 8 Ematologia Ed Immunologia Clinica, Clinica Medica Generale, University of Ancona, Ancona, Italy; 9 Clinical Experimental Onco-Haematology Unit, Centro di Riferimento Oncologico di Aviano (CRO) IRCCS, 33081 Aviano, Italy; 10 UOS Di Immunologia Clinica, Ospedale S. Carlo Borromeo, Milan, Italy; 11 Unità Di Reumatologia, Ospedale Niguarda Ca’ Granda, Milan, Italy; 12 Department of Translational and Precision Medicine, Sapienza University of Rome, Rome, Italy; 13 UO Reumatologia, Ospedale M. Scarlato, Scafati, Salerno, Italy; 14 Department of Internal Medicine, Rheumatology Unit, University of Modena and Reggio Emilia, Modena, Italy; 15 Medicina Interna, University of Florence, Florence, Italy; 16 Medicina Interna, DIMO, University of Bari, Bari, Italy; 17 Department of Medicine, Surgery and Dentistry, Medicina Interna, Policlinico San Marco of Zingonia, University of Milan, Milan, Italy; 18 Clinica Ematologica, AO San Gerardo, University of Milan — Bicocca, Monza, Italy; 19 Infectious Disease Unit, L. Sacco, Department of Clinical Sciences, University of Milan, Milan, Italy

**Keywords:** Cryoglobulins, Mixed cryoglobulinemic syndrome, Cryoglobulinemic vasculitis, HCV, Rituximab, Recommendations, Consensus

## Abstract

Cryoglobulinemic vasculitis (CV) or mixed cryoglobulinemic syndrome (MCS) is a systemic small-vessel vasculitis characterized by the proliferation of B-cell clones producing pathogenic immune complexes, called cryoglobulins. It is often secondary to hepatitis C virus (HCV), autoimmune diseases, and hematological malignancies. CV usually has a mild benign clinical course, but severe organ damage and life-threatening manifestations can occur. Recently, evidence in favor of rituximab (RTX), an anti-CD 20 monoclonal antibody, is emerging in CV: nevertheless, questions upon the safety of this therapeutic approach, especially in HCV patients, are still being issued and universally accepted recommendations that can help physicians in MCS treatment are lacking. A Consensus Committee provided a prioritized list of research questions to perform a systematic literature review (SLR). A search was made in Medline, Embase, and Cochrane library, updated to August 2021. Of 1227 article abstracts evaluated, 27 studies were included in the SLR, of which one SLR, 4 RCTs, and 22 observational studies. Seventeen recommendations for the management of mixed cryoglobulinemia with rituximab from the Italian Study Group of Cryoglobulinemia (GISC) were developed to give a valuable tool to the physician approaching RTX treatment in CV.

## Introduction

Mixed cryoglobulinemia (MC) is the clinical condition resulting from the proliferation of B-cell clones producing pathogenic immune complexes, called type-II and type-III cryoglobulins [1]. Mixed cryoglobulins are often secondary to hepatitis C virus (HCV) and other infective agents, or autoimmune diseases, like Sjögren’s syndrome (SjS) and systemic lupus erythematosus (SLE) [2]. Cryoglobulinemic vasculitis (CV) is a systemic small-vessel vasculitis due to cryoglobulin-containing immune complexes. The term mixed cryoglobulinemia syndrome (MCS) refers to the clinical manifestations that include skin, joints, peripheral nervous system (PNS), and kidneys involvement; rarely also lungs, gastrointestinal tract, and cardiac manifestations are reported [3]. Usually, MCS has a mild benign clinical course, but severe organ damage and life-threatening manifestations can occur [4]. Recently, evidence in favor of anti-CD20 monoclonal antibody treatment with rituximab (RTX) is emerging in MCS [4, 5], but questions upon the safety of this therapeutic approach, especially in HCV patients [6], are still being issued and universally accepted recommendations that can help physicians in MCS treatment are lacking. Through a systematic literature review (SLR) and a subsequent consensus conference, we developed a set of recommendations that we suggest as a valuable tool for the physician approaching RTX treatment in MCS.

## Methods

### Systematic literature review

The research question of the present SLR aimed to look at the benefits and harms of rituximab in mixed cryoglobulinemia (both infectious and non-infectious). The question was rephrased according to the PICOS methodology. We included articles in English concerning adult participants with infectious and non-infectious type II MCS treated with RTX for major (glomerulonephritis, peripheral neuropathy, cutaneous vasculitis) and minor clinical indications (purpura, arthralgia, asthenia). For the intervention, we considered RTX alone or in combination versus placebo or another intervention, including the first line, subsequent treatment lines, or re-treatment. Studies with various dosing schedules and follow-up (short, 6 months; long, greater than 6 months) were also selected. The papers included SLR, randomized controlled trials (RCTs), observational studies (prospective and retrospective cohort and case–control studies), and case series of at least five patients. Medline (via PubMed), Embase, and Cochrane Central were searched until August 2021. In detail, the search strategy adopted to perform the SLR in the three databases included the following terms: for MEDLINE (via Pubmed) (“cryoglobulinemia”[MeSH Terms] OR “cryoglobulinaemia”[All Fields] OR “cryoglobulinemia”[All Fields]) AND (“rituximab”[MeSH Terms] OR “rituximab”[All Fields]); for Embase (“cryoglobulinemia”/exp OR “cryoglobulinaemia” OR “cryoglobulinemia” OR “cryoimmunoglobulinaemia” OR “cryoimmunoglobulinemia” OR “mixed cryoglobulinemia” OR “mixed cryoglobulinemia vasculitis”/exp) AND “rituximab”/exp and (“cryoglobulinemia” or “cryoglobulinaemia”) AND rituximab for Central. The final list of the included studies was evaluated by the expert panel who validated the strategy and reported any relevant references not included in the SLR. In the first step, the selection of studied was based on titles and abstracts. Two authors (AB and AM) independently assessed retrieved abstracts and, if necessary, the full text of these studies to determine which papers satisfied the inclusion criteria. Disagreement regarding the inclusion of an article was discussed between reviewers until consensus was reached. Persistent disagreements were resolved by a third evaluator (LQ). Data extraction was carried out independently by two authors using standard data extraction forms. The results of the SLR were sent to the committee before the second meeting, together with proposals for recommendations.

### Expert Committee and development of recommendations

A Consensus Committee consisted of 30 physicians working in various medical fields (internal medicine, rheumatology, hematology, nephrology, hepatology, infectious diseases, and neurology, clinical epidemiology) provided a prioritized list of research questions to perform a SLR. The experts were invited to define the coverage of the recommendations including the safety and efficacy of RTX treatment in infectious and non-infectious MCS, which were to be used as search terms for the SLR. Sixteen relevant clinical questions were composed for the SLRs, according to a pre-specified protocol. The recommendations summarized in this article represent a consensus of published evidence and expert opinions. Standard of care in HCV-related, HCV-unrelated, and noninfectious CV is largely based on qualified expert experience and specific literature [7, 8]. For each recommendation, we used a widely-accepted hierarchy for categorizing the available evidence and the strength of the recommendations (evidence categories A–D) (Tables 1 and 2). Specific recommendations were separately voted and scored from 0 (no agreement with) to 100 (maximal agreement). The means and SD of the scores were calculated to determine the level of agreement among the experts’ panel for each recommendation. Total cumulative agreement ≥ 70 defined consensus for each statement. In the case of lack of agreement, the statement was reworded according to the results of the discussion and then re-voted. Then the level of evidence (LoE) was assigned.

**Table 1:** Evidence categories and strength of recommendations [9]

Category	Evidence
Ia	Meta-analysis of randomized controlled trials
Ib	Randomized controlled trial
II	Prospective controlled intervention study without randomization
III	Prospective cohort studies
IV	Case-series, poor quality cohort and case–control studies
V	Expert committee reports or opinion or clinical experience of respected authorities or both
Strength	Based on
A	Consistent level I studies
B	Consistent level II or III studies or extrapolated recommendations from level 1 studies
C	Level IV studies or extrapolated recommendations from level II or III studies
D	Level V evidence

**Table 2:** The final set of recommendations for the management of mixed cryoglobulinemia with rituximab from the Italian Study Group of Cryoglobulinemia (GISC)

Statements	Mean	Standard deviation
1. Overall, rituximab is effective (and safe) on the severe, not immediately life-threatening, clinical manifestations of cryoglobulinemic vasculitis (LoE 1A)	92,33	7,42
2. In particular, rituximab is effective (and safe) on the glomerulonephritis of cryoglobulinemic vasculitis (LoE 2B)	91,92	8,62
3. In particular, rituximab is effective (and safe) on the peripheral neuropathy of cryoglobulinemic vasculitis (LoE 2C)	77,71	14,51
4. In particular, rituximab is effective (and safe) on the skin ulcers of cryoglobulinemic vasculitis (LoE 1A)	85,21	13,08
5. Rituximab is equally effective on other, not severe manifestations (purpura, arthralgia, fatigue) of cryoglobulinemic vasculitis (LoE 2B)	80,00	16,39
6. Rituximab may be equally effective in infectious and non-infectious cryoglobulinemic vasculitis (LoE 5C)	76,92	16,69
7. Rituximab should be used cautiously in patients carrying latent HBV infection, provided that and an adequate prophylactic therapy for HBV infection, or monitoring HBV DNA or HBsAg title should be done (LoE 5C)	92,42	26,54
8. Re-treatment at clinical relapse with rituximab, after the first cycle, is effective (and safe) in patients with severe, not immediately life-threatening, clinical manifestations of cryoglobulinemic vasculitis (LoE 2B)	90,17	10,71
9. Rituximab shows a “steroid-sparing” effect in patients with severe, not immediately life-threatening, clinical manifestations of cryoglobulinemic vasculitis (LoE 2B)	92,17	8,68
10. Rituximab does not usually carry an increased risk of serious adverse events compared to other immunosuppressants or high-dose glucocorticoids. Attention should be paid for repeated courses and multiple comorbidities (LoE 1,A)	90,31	21,17
11. Rituximab given alone is not associated with an increased risk of hepatitis C reactivation, even if a transient elevation of the viral load can be seen (LoE 1B)	89,50	19,68
12. The risk of severe infusion reactions during rituximab administration is very low (LoE 1A)	87,58	10,94
13. Rituximab is effective and safe in combination with antivirals in some cases of cryoglobulinemic vasculitis (LoE 5C)	91,38	11,55
14. Rituximab is effective in patients with HCV-related cryoglobulinemic vasculitis showing persistent and severe clinical course, despite virological clearance by antivirals (LoE 5C)	89,92	9,05
15. Rituximab given at low doses (250 mg/mq weekly for 2 weeks) is equally effective as given at high doses (375 mg/mq/weekly for 4 weeks or 1 g 2 weeks apart) in somecases of cryoglobulinemic vasculitis (LoE 5C)	72,00	27,16
16. Maintenance treatment with rituximab is required in severe or life-threatening cryoglobulinemic vasculitis (LoE 5C)	74,58	29,47

## Results

**Fig. 1:**
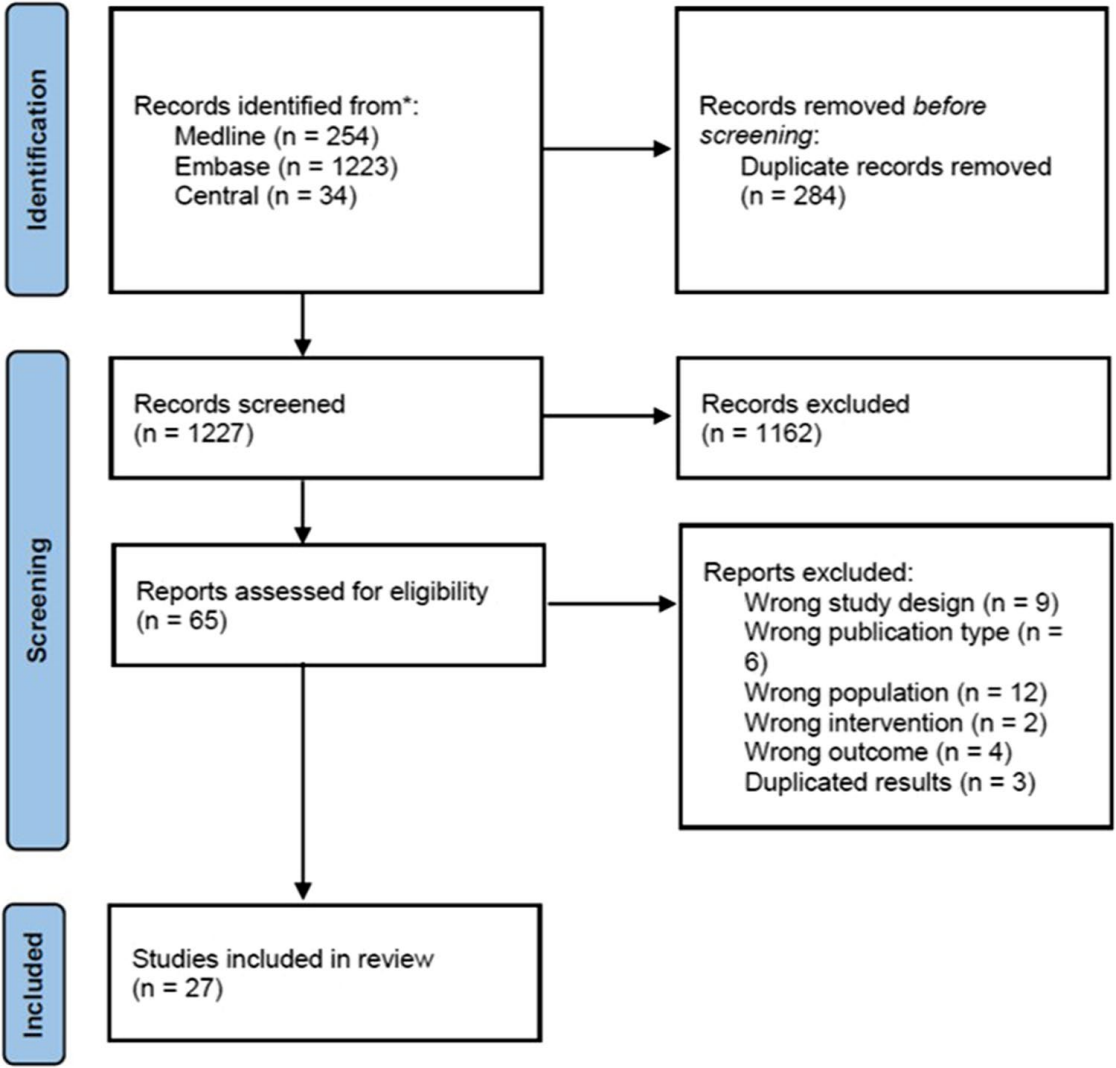
PRISMA 2020 flow chart showing the studies selection

Of 1227 article abstracts evaluated, 27 studies were included in the SLR (Fig. 1), of which one SLR, 4 RCTs, and 22 observational studies.

### Recommendations

#### 1. Overall, rituximab is effective (and safe) on the severe, not immediately life-threatening, clinical manifestations of cryoglobulinemic vasculitis (LoE 1A)

Moderate to severe manifestations of MCS comprise glomerulonephritis, digital ischemia or necrotizing skin ulcers, polyarthritis, gastrointestinal vasculitis, and neuropathy.

For most patients with moderate to severe MCS, RTX is considered the treatment of choice whenever it is necessary to contain the proliferation of the cell clones responsible to produce cryoglobulins. In severe manifestations of MCS, evidence supporting the effectiveness of RTX compared with non-RTX treatment comes from one Cochrane SLR including three randomized controlled trials in MCS induced by HCV infection for a total of 118 participants [10–13]. Dammacco in the 2010 study included antiviral treatment in both groups, while in the other two studies, patients’ inclusion implied that therapy with antiviral agents had failed, had been poorly tolerated, or contraindicated [10, 11]. Additional observational studies supported the efficacy of RTX in patients with moderate or severe MCS not associated with chronic HCV infection [5, 14–19].

The indication for the use of RTX in severe and life-threatening MCS is reported in a separate core set of recommendations [3].

#### 2. In particular, rituximab is effective (and safe) on the glomerulonephritis of cryoglobulinemic vasculitis (LoE 2B)

Glomerulonephritis is the most common form of renal involvement in MCS and represents a harmful complication. Prevalence of glomerulonephritis in MCS patients ranges between 20 and 30% and when present, a three-fold increase in the mortality risk is observed [3, 20]. Treatment with RTX has proven to be effective in cryoglobulinemic glomerulonephritis. In the RCT conducted by Sneller et al., 4 cases of glomerulonephritis treated with RTX achieved a stable renal function or improvement in the estimated glomerular filtration rate (eGFR), while patients in the control group treated with immunosuppressive agents had a decline in the eGFR [13]. Several observational studies highlighted high rates of clinical remission, with consistent improvement in renal function. Normalization of active urinary sediment and reduction of proteinuria was frequently observed [5, 19, 21–24].

#### 3. In particular, rituximab is effective (and safe) on the peripheral neuropathy of cryoglobulinemic vasculitis (LoE 2C)

PNS involvement is frequent in MCS, often presenting as peripheral polyneuropathy. Symptoms such as paresthesia and pain are present in up to 90% of MCS patients, while nearly 80% of them have abnormal electromyography (EMG) findings [25]. Evidence supporting RTX as an effective treatment in PNS involvement can be extracted from one RCT that included 16 participants with PNS involvement randomized at RTX administrations versus 17 patients treated with non-RTX therapy, consisting of glucocorticoids, azathioprine, cyclophosphamide, or plasmapheresis [10]. Twelve out of 14 patients experienced a clinical improvement expressed in terms of visual analogical scale (VAS) pain and VAS paresthesia at 12 months, proving non-inferiority to the control arm [10].

Additionally, several retrospective studies and case series reported the efficacy of RTX in PNS involvement, inducing clinical and EMG improvement [5, 19, 22, 24, 26–28]. Evidence supported the efficacy of RTX in preventing the progression of active MCS neuropathy, while data suggest a limited value in recovering from acquired and chronic damage. This aspect induced the experts to emphasize the early use of RTX in the active form of PNS involvement.

#### 4. In particular, rituximab is effective (and safe) on the skin ulcers of cryoglobulinemic vasculitis (LoE 1A)

Necrotizing skin ulcers are non-healing cutaneous lesions, occurring in 25–30% of MCS patients. These lesions are possibly complicated by local infection and gangrene, worsening patients’ quality of life and overall prognosis [29]. In this scenario, B-cell depletion represents a valid choice of treatment for severe skin ulcers. One Cochrane SLR including two RCTs for a total of 78 patients with HCV-related MCS highlighted that RTX may improve skin manifestations, including vasculitis and ulcers, at 18–24 months compared to controls (*RR* 0.57, 95% *CI* 0.28 to 1.16) [11, 12]. Observational studies including a proportion of patients with non-HCV related MCS reported high rates of complete response to RTX treatment for skin ulcers [5, 19, 22, 24, 28].

#### 5. Rituximab is equally effective on other, not severe manifestations (purpura, arthralgia, fatigue) of cryoglobulinemic vasculitis (LoE 2B)

Multiple observational studies included the evaluation of the clinical response to treatment for mild clinical manifestations such as purpura, arthralgia, and fatigue as outcome measures. Data from these studies conducted in a mixed cohort of HCV and non-HCV-related MCS provided evidence that RTX treatment was effective for mild manifestations, resulting in a rapid improvement of clinical signs in the majority of patients with a positive impact on the patient’s VAS pain assessment [5, 17, 21, 22, 24, 28].

#### 6. Rituximab is equally effective in infectious and non-infectious cryoglobulinemic vasculitis (LoE 5C)

The majority of MCS patients are HCV positive; however, non-infectious CV can be observed, particularly in connective tissue diseases such as SjS and SLE [1]. Most of the studies tested RTX in cohorts of HCV-related MCS patients [11, 13] or were conducted in mixed cohorts of infectious and non-infectious MCS patients [10, 16, 27, 30]. Only limited data are available on RTX treatment in HCV negative MCS subjects. Based on the available evidence, including case reports and common clinical practice, the expert panel agreed that RTX is equally effective in infectious and non-infectious CV.

#### 7. Rituximab should be used cautiously in patients carrying latent HBV infection, provided that an adequate prophylactic therapy for HBV infection, or monitoring HBV DNA or HBsAg title should be done (LoE 5C)

B-cell depleting therapies are associated with an increased risk of hepatitis reactivation in both HBsAg-positive as well as in HBsAg-negative and anti-HBc–positive patients [31, 32]. HBV reactivation in the setting of immunosuppression related to RTX treatment is a potentially life-threatening condition leading to liver failure and death in extreme cases if it is not promptly recognized [32]. In MCS, the expert committee highlighted to use of RTX cautiously in patients carrying latent HBV infection. An adequate assessment of MCS patients is mandatory, including monitoring of HBsAg title and HBV DNA during RTX treatment and in follow-up. Institution of prophylactic antiviral therapy before initiation of immunosuppression is essential.

#### 8. Re-treatment at clinical relapse with rituximab, after the first cycle, is effective (and safe) in patients with severe, not immediately life-threatening, clinical manifestations of cryoglobulinemic vasculitis (LoE 2B)

Relapses occur frequently during the course of MCS, contributing prominently to disease burden and mortality. Severe, not immediately life-threatening, relapses can be managed with RTX re-treatment. Several observational studies proved that repeated courses of RTX are effective and safe: Quartuccio et al. observed complete remission in one third (6/17, 35.3%), partial response in 5/17 (29.4%), and no response in 6/17 of the retreated cases with a mean time to retreatment of 22.3 (± 12.1) months [33]. Comparable results, with an overall response rate of approximately 66%, are reported by other observational and retrospective studies [10, 18, 19, 30].

In addition, no differences in terms of efficacy and safety were highlighted between different RTX regimens used for the retreatment: Quartuccio et al. used a dosage of 1000 mg/m2 given twice, while Colantuono et al. administered low dose RTX 250 mg/m2 twice 1 week apart — with comparable results (remission achieved in 2/3 of the patients) [30, 33].

#### 9. Rituximab shows a “steroid-sparing” effect in patients with severe, not immediately life-threatening, clinical manifestations of cryoglobulinemic vasculitis (LoE 2B)

Corticosteroids are historically considered the first-line treatment in MCS, with great efficacy in severe and moderate clinical manifestations. However, long-term exposure to corticosteroids carries a high burden of important side effects, thus minimization of the dose should be encouraged. Evidence for the efficacy of B-cell depletion therapy as a steroid-sparing agent can be extrapolated from Sneller et al. In this RCT, 6 patients with HCV-related MCS received corticosteroids at study entry in the RTX arm compared with 3 HCV-related MCS patients treated with immunosuppressive therapy in the control group [13]. Five of them discontinued steroids during the study, and one patient maintained low dosage of prednisone to prevent adrenal insufficiency [13]. Additional evidence supporting RTX efficacy in steroid tapering comes from an observational study involving a cohort of 15 patients with HCV- and non-HCV-related MCS, treated with RTX 375 mg/m2 1 week apart for four times. In this study, 8 patients were under corticosteroid treatment at the time of enrolment and 7 of them reduced and achieved steroid-free treatment shortly after RTX initiation [24].

#### 10. Rituximab does not usually carry an increased risk of serious adverse events compared to other immunosuppressants or high-dose glucocorticoids. Attention should be paid for repeated courses and multiple comorbidities (LoE 1, A)

Immunosuppressive treatment, while highly effective, always generates doubts and concerns in terms of safety, especially in fragile patients. RTX treatment is not associated with an increased risk of serious adverse events, as recently highlighted by a Cochrane SLR. Statistical analyses conducted on data from three RCTs including 118 patients with HCV-related MCS did not show differences between RTX and control groups in terms of discontinuation of treatment due to adverse reactions (*RR* 0.97, 95% *CI* 0.22 to 4.36) [11]. The infective risk was analyzed in two RCTs for a total of 83 patients: no differences between RTX and control group were found. Moreover, several observational studies confirmed the good safety profile of RTX in MCS, including a small cohort of non-HCV-related MCS, without a significant increase in serious adverse events [5, 10, 17, 18, 22, 24, 26].

Only one monocentric observational study raised doubts on RTX safety, reporting a 27.3% incidence of serious adverse events in a cohort of 22 patients. Further analysis showed that RTX regimen 375 mg/m^2^/week for 4 consecutive weeks was associated with more frequent serious adverse events than treatments with lower dosages (1 g 2 weeks apart) (50% versus 6.25%; *P*: 0.046) [34]. The expert panel recommended caution in repeated RTX courses and in patients with multiple comorbidities, particularly cardiovascular diseases, that represent a well-known potential side effect of this class of drugs.

#### 11. Rituximab given alone is not associated with an increased risk of hepatitis C reactivation, even if a transient elevation of the viral load could be seen (LoE 1B)

HCV reactivation following B cell depletion is a concerning issue, especially after the publication of some observational studies that highlighted the increase of HCV-RNA levels in patients treated with RTX [3, 25]. To address this problem, Dammaco et al. treated patients with a combined RTX-ribavirin-IFN regimen, observing no clinical hepatitis reactivation [11]. In two RCTs, involving 78 patients with HCV-related MCS under RTX treatment, no difference in the risk of hepatitis reactivation was reported between the interventional arm and patients treated with conventional immunosuppressive therapy in control groups [10, 13]. In detail, De Vita et al. reported no significant increase in liver enzyme levels in the RTX and the control group (*P*: 0.868 for the RTX group; *P*: 0.538 for the non-RTX group); similarly, Sneller et al. showed that no patient in the RTX group developed clinical or laboratory evidence of worsening hepatic function [10, 13].

Many other observational studies reported similar results, providing additional evidence for RTX as a safe therapeutic approach in HCV patients [14, 18, 21, 22, 24, 28, 33]. To date, no data are available for RTX administered in combination with new direct-acting antiviral agents (DAAs).

In conclusion, although a transient elevation in serum HCV-RNA can be seen in HCV patients treated with RTX as a monotherapy regimen [24, 28], no increased risk of clinical re-activation merged from current data.

#### 12. The risk of severe infusion reactions during rituximab administration is very low (LoE 1A)

Both cytotoxic and monoclonal antibodies are associated with an increased risk of infusion reactions: RTX in particular has been associated with IgE-mediated allergic reactions and cytokine-release reactions due to the cytokine released from the binding of the drug to the target cells [35, 36]. Both these mechanisms of action are implicated in infusion reactions, which are defined as mild when clinical manifestations like chills, fever, mild hypotension, dyspnea, and rash are present, or severe when hypotension anaphylaxis and cardiac dysfunction occur [36]. For this reason, RTX is usually administered following a strict infusion protocol including a premedication with parenteral antihistaminic and corticosteroids [10, 11, 36]. A Cochrane SLR including 3 RCTs for a total cohort of 118 MCS patients treated with RTX reported a slight increase in infusion reactions compared to other immunosuppressive medications (*RR* 4.33, 95% *CI* 0.76 to 24.75) [12]. However, there was little or no difference in discontinuation of treatment due to adverse reactions (*RR* 0.97, 95% *CI* 0.22 to 4.36) and only one patient developed a severe infusion reaction (fever to 40.5 °C, resolved within 1 h) in the total cohort of 118 patients treated with RTX [10–13]. Additionally, one large observational study including 31 MCS patients treated with B-cell depletion reported no infusion reactions [22].

#### 13. Rituximab is effective and safe in combination with antivirals in some cases of cryoglobulinemic vasculitis (LoE 5C)

Focusing on HCV-related MCS, combination therapy of RTX and antivirals has a potential role in clinical practice. Data supporting its effectiveness and safety can be extracted from Dammacco et al. In this RCT, 22 patients were treated with IFN/ribavirin/RTX regimen and about 50% of them showed a complete response to therapy and no serious adverse events were recorded [11].

Combination therapy with B-cell depletion and antivirals resulted effective and safe also in a cohort of 20 patients followed in an observational study, with a large number of complete responders and no serious adverse events [18]. Currently, no strong data are available for DAAs, but the expert committee expressed a positive opinion for the employment of DAAs in association with RTX.

#### 14. Rituximab is effective in patients with HCV-related cryoglobulinemic vasculitis showing persistent and severe clinical course, despite virological clearance by antivirals (LoE 5C)

B cell proliferation could become trigger-independent and lead to the persistence of CV manifestations after viral eradication in patients with HCV-related MCS [37, 38]. RTX stands as a possible therapeutic approach for those situations, as stated in “6” for non-HCV MCS.

Currently, no RCTs or observational studies including HCV-related MCS patients that achieved HCV-RNA clearance are available; however, the expert committee suggested RTX treatment for the management of these patients. In conclusion, only current clinical practice supports at present the effectiveness of B-cell depletion in HCV-related CV showing persistent and severe clinical course despite virological clearance by antivirals.

#### 15. Rituximab given at low doses (250 mg/mq weekly for 2 weeks) is equally effective as given at high doses (375 mg/mq/weekly for 4 weeks or 1 g 2 weeks apart) in some cases of cryoglobulinemic vasculitis (LoE 5C)

RTX high dosage consisting of 375 mg/m2 weekly for 4 weeks, the therapeutic regimen utilized in non-Hodgkin lymphomas, was used in the large majority of studies in MCS conducted in the past, including two previously discussed RCTs [11, 13]. Recently, Visentini et al. have proposed a low dose RTX regimen of 250 mg/mq weekly for 2 weeks in a phase II clinical trial involving 52 MCS patients with severe manifestations. Forty-one of 48 evaluable patients (85%) achieved a clinical response with a median time to remission/improvement of vasculitis of 1 month [15]. Later, Colantuono et al. have utilized the same low dose RTX regimen in an observational study, treating 37 MCS patients with a response rate of 80%, and with complete remission in 68% of patients [30]. Another observational study involving 31 MCS patients treated with RTX 250 mg/mq weekly for 2 weeks reported a complete clinical response in 22 subjects (70.96%) [39]. No head-to-head trials comparing different RTX regimens are currently available; however, given these previously mentioned positive results, the expert committee stated that both low-doses and high-doses of RTX regimens can be considered equally effective in selected cases of CV. The Italian Medicines Agency officially endorsed this regimen in 2020 [40]. The experts agreed that high-doses of RTX should be preferred in severe manifestations such as rapidly progressive glomerulonephritis or acute motor neuropathy, as well as life-threatening conditions, such as alveolar hemorrhage, intestinal vasculitis, or central nervous system vasculitis.

#### 16. Maintenance treatment with rituximab is required in severe or life-threatening cryoglobulinemic vasculitis (LoE 5C)

As Pietrogrande et al. previously stated in a set of recommendations for managing HCV-related MCS, there are still open questions on maintenance treatment with RTX [8]. Evidence in favor of B-cell depletion as maintenance therapy comes from ANCA (anti-neutrophil cytoplasmic antibody) associated vasculitis, where RCTs demonstrated the superiority of RTX versus conventional immunosuppressants for this purpose [41, 42]. For MCS, maintenance therapy with RTX is rarely described; however, some reports showed that it can be considered in severe cases of nephritis and abdominal vasculitis [43, 44]. In addition, as previously debated, retreatment with RTX has proven to be effective and safe in MCS, thus, the expert committee stated that, given the positive risk/benefit ratio, maintenance treatment with RTX is required in severe or life-threatening CV.

## Discussion

Cryoglobulinemic vasculitis (CV) or mixed cryoglobulinemic syndrome (MCS) is classified among the group of vasculitides affecting small vessels [45]. The most frequent etiology is the HCV infection, followed by SjS and hematologic malignancies [46, 47].

However, in the last 5 years, the introduction of the new DAAs is rapidly decreasing the HCV-related CV in favor of other etiologies [48].

Nevertheless, B-cell abnormal activation downstream of the trigger drives the pathogenesis of CV and is the main biological target [49].

RTX, a chimeric monoclonal antibody that targets CD20 positive B cells, has been employed successfully in many systemic autoimmune diseases [50, 51], including ANCA-associated vasculitides [52], in which it is now the worldwide accepted alternative to cyclophosphamide in the induction phase of the treatment. Notably, the first cases of CV treated with RTX [53] date back to 1999 and anticipate a few years the administration of RTX to the first case of granulomatosis with polyangiitis [54].

The placement of RTX in the different clinical scenarios of CV was discussed in the 2011 Consensus Conference of GISC in the wider context of the treatment recommendations for CV [8]. The role of RTX was proposed for patients with severe CV according to published works [21, 26, 55–61] and the single RCT available in abstract form [62].

As a matter of fact, in 2011, it was clearly stated that further investigations, especially RCT, were strongly needed in order to provide further data on several open issues on RTX management in CV: more solid clinical criteria for the application, combination with antiviral therapy, steroid sparing effect, effects on liver function and immune response, duration of response, and re-treatment and maintenance strategies were necessary.

For this reason, the most recent consensus conference has focused on the efficacy and safety of RTX in CV on the basis of a preliminary meta-analysis on the usefulness of RTX in CV. Most of these issues have been addressed in several relevant works published in the last years [5, 15, 16, 19, 22, 23, 27, 30, 33, 39] and three RCTs were conducted [10, 11, 13], finally bringing important evidence-based support to the actual consensus conference. However, most of the trials were not primarily focused on the treatment in study (RTX), and, therefore, this observation represents a limitation of our consensus and it affected the LoE.

Firstly, even if the number of RCTs comparing RTX with the standard of care is still low [10, 13], several observational studies also support the notion that RTX is effective and safe in patients with major clinical manifestations of CV, not immediately life-threatening [5, 13–16, 18, 19, 22].

The management of both severe CV manifestations, such as glomerulonephritis [5, 13, 19, 21–24], skin ulcers [5, 10, 19, 22, 24, 28] and sensory-motor progressive peripheral neuropathy [5, 10, 19, 22, 24, 26–28], and minor manifestations such as purpura, arthralgia, and fatigue [5, 17, 21, 22, 24, 28] were evaluated, resulting in favor of RTX effectiveness together with an acceptable safety profile. Hence, the use of RTX has gained more strength based on the above-mentioned works, showing a higher level of evidence (LoE) of actual statements concerning RTX, compared to the previous recommendations (from LoE 3C for the single statement only available in 2011 to actual several and more detailed statements, respectively: LoE 1B concerning the overall statement, LoE 1B concerning in particular the efficacy on skin ulcers; LoE 2C concerning the efficacy on glomerulonephritis and peripheral neuropathy, LoE 2B concerning the minor manifestations). However, the heterogeneity of the works, the low number of RCTs, and the low number of patients included did not allow the most recent published meta-analyses on CV manifestations, especially for renal involvement [12], or CV neuropathy [63] to support the efficacy of RTX in CV observed in observational studies so far [4]. The absence of a standardized response criteria in CV, and in particular in the renal involvement, could greatly affect the final conclusions and it represents an important unmet need for the research agenda in CV.

Importantly, it is widely agreed that low-dose regimen is equally effective than high-dose regimen of RTX in CV [14, 15, 18, 30, 39], even if life-threatening manifestations (such as intestinal vasculitis or alveolar hemorrhage) could require the high-dose regimen, even in combination with cyclophosphamide or plasma exchange [4].

Moreover, the role of RTX as steroid sparing agent in patients with severe clinical manifestations was confirmed [10, 13, 24]: therefore, RTX can be considered a valid approach to greatly cut down the long-term administration of low-to-medium dose of glucocorticoids. This topic was widely faced also in the previous consensus conference of 2011, in which glucocorticoid-related side effects were highlighted, considering the older age of many patients, the high incidence of co-morbidities, the concomitant chronic HCV or HBV infection, and the risk of other infections. In fact, the statement in the actual consensus conference gained a higher LoE (from LoE 3C to LoE 2B).

As concerns subsequent cycles of RTX, RTX re-treatment after the first cycle appeared successful and safe in patients with major relapse [10, 18, 19, 30, 33]. Moreover, maintenance with RTX can be required in severe or life-threatening CV [4]. Monitoring the serum level of immunoglobulins is advisable in this scenario for the risk of hypogammaglobulinemia and infections.

As far as safety outcomes are concerned, RTX does not increase the risk of clinical reactivation of hepatitis in HCV-related CV [10, 13, 14, 18, 21, 22, 24, 33]; however, regarding viral load, even if RTX was not associated with increasing viral load in most papers [13, 14, 17, 18, 22, 26, 57], a transient not clinically relevant increase in HCV viral load can occur [21, 28]. By contrast, the management of HBV-related CV requires more caution by the clinicians given the occurrence of serious HBV reactivation under B-cell depleting therapy [64]. Overall, RTX does not increase the risk of serious adverse events [5, 10, 17, 18, 22, 24, 26], including infusion reactions [11, 13, 22]. Importantly, previous treatments with corticosteroids and/or cyclophosphamide can increase the risk of superimposed infections under RTX [10]. Nevertheless, data from the French AutoImmunity and Rituximab (AIR) registry showed the occurrence of severe infections in the elderly, with essential type II mixed cryoglobulinemia and renal failure with a glomerular filtration rate lower than 60 ml/min, and receiving high-dose corticosteroids [65].

Yet, the French group suggested caution in a subgroup of CV, in which RTX could determine severe infusion reaction [34], or even RTX-associated immune complex vasculitis [66]. Since RTX-associated cryoglobulinemia vasculitis flare has been linked to high mortality rate and resistance to corticosteroids and/or plasma exchange, clinicians should consider the following risk factors for this dangerous condition: renal vasculitis, B cell lymphoma, higher level of cryoglobulin, and lower level of C4. The correct application of the protocol of premedication before RTX infusions including intravenous methylprednisolone (100 mg), intravenous chlorpheniramine maleate (10 mg), and paracetamol (1 g), and eventually the use of plasma exchange before RTX [34, 66], or the lower doses of RTX, if appropriate, might mitigate this complication.

RTX can be used in combination with antivirals in HCV-related CV [11, 18]. Additionally, it is effective in patients with persistent and severe CV despite viral clearance [10, 13–15, 17, 18, 21, 22, 24, 28]. This observation is of particular relevance, even in the era of DAAs, the most effective drugs against HCV, because cryoglobulin production can persist after HCV eradication in about 50% of patients [67], and also CV can persist or relapse even after HCV disappearance [68].

Unfortunately, no sufficient data were available to declare that RTX could be effective in non-infectious CV as in HCV-related ones. Skin manifestations were reported to be more susceptible to RTX than neuropathy or glomerulonephritis in noninfectious CV [69]. However, safety issue is a matter of concern in this setting, especially for the risk of superimposed infections [70].

Pharmacoeconomics is a pressing topic nowadays, then biosimilars of RTX are worldwide applied in all approved indications [71] and even in the off-label use [72].

RTX biosimilar CT-P10 has been approved in Europe. One recently published observational study analyzed 51 MCS patients treated with CT-P10 in the interventional arm, compared with a retrospective group of 75 MCS patients treated with RTX originator. No significant difference between the two groups emerged in terms of severe adverse events and clinical efficacy [73]. Supported by this positive result, CT-P10 may replace the RTX originator, considering its cost/efficacy ratio.

Very recently, combining different B-cell targeting therapies is becoming a new treatment strategy with preliminary success in some autoimmune diseases, including SjS. Also, this treatment approach may be effective even in refractory cases of CV [74–76].

In conclusion, for over two decades, RTX has been successfully employed in many people suffering from hematological malignancies and many different autoimmune diseases, and it has been finally stated in the WHO Model List of Essential Medicines which should be available in all healthcare systems. CV is a rare systemic vasculitis, in which an increasing amount of knowledge has been provided since the discovery of the HCV as the main etiology in the 1990. The scientific effort of the last years allowed us to generate new statements with stronger LoE regarding the efficacy and safety of RTX in CV, now placing RTX together with DAAs, for HCV-related CV, as fundamental therapy for the management of people suffering from CV. As monotherapy or in combination with other treatments, by applying low-dose regimen or high-dose regimen, RTX has been proved effective in the whole spectrum of CV manifestations from the mildest to the most severe ones, thus now representing the backbone immunosuppressive treatment of CV. Importantly, the optimal use of RTX in CV should be tailored on each case by the expert clinician, since the number of studies with the highest level of evidence is still low. Yet, the level of evidence on the efficacy and safety of RTX in noninfectious CV is still low and requires further larger studies. Finally, improvement of RTX efficacy in CV by adding other B-cell targeting therapies seems to be promising in refractory cases of CV, in particular in SjS-related CV.
