# Correction to: Management of mixed cryoglobulinemia with rituximab: evidence and consensus-based recommendations from the Italian Study Group of Cryoglobulinemia (GISC)

**DOI:** 10.1007/s10067-022-06414-6

**Published:** 2022-10-20

**Authors:** Luca Quartuccio, Alessandra Bortoluzzi, Carlo Alberto Scirè, Antonio Marangoni, Giulia Del Frate, Elena Treppo, Laura Castelnovo, Francesco Saccardo, Roberta Zani, Marco Candela, Paolo Fraticelli, Cesare Mazzaro, Piero Renoldi, Patrizia Scaini, Davide Antonio Filippini, Marcella Visentini, Salvatore Scarpato, Dilia Giuggioli, Maria Teresa Mascia, Marco Sebastiani, Anna Linda Zignego, Gianfranco Lauletta, Massimo Fiorilli, Milvia Casato, Clodoveo Ferri, Maurizio Pietrogrande, Pietro Enrico Pioltelli, Salvatore De Vita, Giuseppe Monti, Massimo Galli

**Affiliations:** 1 Unit of Rheumatology, Department of Medicine (DAME), University of Udine, ASUFC, Udine, Italy; 2 Section of Rheumatology, Department of Medical Sciences, University of Ferrara and Azienda Ospedaliera, Universitaria Di Ferrara, Cona, FE Italy; 3 Epidemiology Unit, Italian Society for Rheumatology (SIR), Milan, Italy; 4 Department of Internal Medicine, Hospital of Legnano, Legnano, Italy; 5 Medicina Interna, Ospedale Di Saronno, AO Busto Arsizio, Italy; 6 UO Nefrologia, Spedali Civili, Brescia, Italy; 7 ASUR Marche ZT6, Fabriano, Italy; 8 Ematologia Ed Immunologia Clinica, Clinica Medica Generale, University of Ancona, Ancona, Italy; 9 Clinical Experimental Onco-Haematology Unit, Centro di Riferimento Oncologico di Aviano (CRO) IRCCS, 33081 Aviano, Italy; 10 UOS Di Immunologia Clinica, Ospedale S. Carlo Borromeo, Milan, Italy; 11 Unità Di Reumatologia, Ospedale Niguarda Ca’ Granda, Milan, Italy; 12 Department of Translational and Precision Medicine, Sapienza University of Rome, Rome, Italy; 13 UO Reumatologia, Ospedale M. Scarlato, Scafati, Salerno Italy; 14 Department of Internal Medicine, Rheumatology Unit, University of Modena and Reggio Emilia, Modena, Italy; 15 Medicina Interna, University of Florence, Florence, Italy; 16 Medicina Interna, DIMO, University of Bari, Bari, Italy; 17 Department of Medicine, Surgery and Dentistry, Medicina Interna, Policlinico San Marco of Zingonia, University of Milan, Milan, Italy; 18 Clinica Ematologica, AO San Gerardo, University of Milan — Bicocca, Monza, Italy; 19 Infectious Disease Unit, L. Sacco, Department of Clinical Sciences, University of Milan, Milan, Italy

**Correction to: Clinical Rheumatology**

10.1007/s10067-022-06391-w

In the original published version of this article, the co-author, Cesare Mazzaro affiliation was incorrect and should have been "Clinical Experimental Onco-Haematology Unit, Centro di Riferimento Oncologico di Aviano (CRO) IRCCS, 33081 Aviano, Italy"

The original article has been corrected.

